# From Hyperendemic to Low Endemicity: The Effect of Hepatitis B Vaccination on HBV and HDV Prevalence in the Brazilian Amazon

**DOI:** 10.3390/pathogens14111089

**Published:** 2025-10-25

**Authors:** Andreza Pinheiro Malheiros, Michele Soares Gomes-Gouvêa, Leidiane Barbosa Ribeiro, Alex Junior Souza de Souza, Raymundo Soares Azevedo, Dickson Ciro Nascimento de Brito, Candida Maria Abrahão de Oliveira, Heloisa Marceliano Nunes, João Renato Rebello Pinho

**Affiliations:** 1 Seção de Hepatologia, Instituto Evandro Chagas, Secretaria de Vigilância em Saúde e Ambiente, Ministério da Saúde, Ananindeua 67030-000, PA, Brazil; dicksonbrito@iec.gov.br (D.C.N.d.B.); candidaoliveira@iec.gov.br (C.M.A.d.O.); heloisanunes@iec.gov.br (H.M.N.); 2 Programa de Pós-Graduação em Virologia, Instituto Evandro Chagas, Secretaria de Vigilância em Saúde e Ambiente, Ministério da Saúde, Ananindeua 67030-000, PA, Brazil; 3 LIM07, Instituto de Medicina Tropical, Departamento de Gastroenterologia, Hospital das Clínicas HCFMUSP, Faculdade de Medicina, Universidade de São Paulo, São Paulo 05403-000, SP, Brazil; michele.gouvea@hc.fm.usp.br (M.S.G.-G.); leidianebr@usp.br (L.B.R.); joao.pinho@einstein.br (J.R.R.P.); 4 Programa de Pós-Graduação em Saúde Única, Universidade Santo Amaro (UNISA), São Paulo 04829-300, SP, Brazil; souzajralex@gmail.com; 5 LIM01, Departamento de Patologia, Faculdade de Medicina, Universidade de São Paulo, São Paulo 05403-000, SP, Brazil; razevedo@usp.br; 6 Laboratório Clínico, Einstein Hospital Israelita, São Paulo 05652-900, SP, Brazil

**Keywords:** hepatitis B, hepatitis D, epidemiology, hepatitis B vaccine, human viral hepatitis

## Abstract

The Amazon Basin was historically hyperendemic for HBV and HDV, associated with severe outcomes like fulminant hepatitis. Brazil initiated its hepatitis B vaccination in 1989. This study assessed the current prevalence in this endemic region to evaluate the impact of vaccination. A cross-sectional population-based survey enrolled 1100 urban and rural residents. HBsAg prevalence was 1.5%, with no cases in individuals under 20 years, demonstrating interrupted vertical and horizontal transmission. Anti-HBc positivity (30.9%) indicated past exposure, predominantly in those over 30 years. Isolated anti-HBc (10.3%) included two occult HBV infections. HDV coinfection occurred in 25% of HBsAg-positive cases, with HDV RNA detected in two. Anti-HDV positivity was exclusive to adults over 30. Vaccination coverage was poorly documented, but 23.7% had protective anti-HBs titers. HBV vaccination has reduced HBsAg prevalence from high to low endemicity in the region, eliminating chronic infections in younger generations. Persistent HDV in older age groups underscores the need for targeted screening. Despite vaccination record gaps, the findings highlight the program’s success in interrupting transmission and support continued efforts toward HBV/HDV elimination.

## 1. Introduction

The Amazon Basin was a highly endemic region for hepatitis B (HBV) and delta (HDV) viruses, with elevated prevalence rates documented across Brazil, Colombia, Venezuela, Peru, and Ecuador [1,2,3,4,5,6]. In these countries, cases of fulminant hepatitis, later attributed to HBV/HDV superinfection or coinfection, were reported. In Brazil, this condition was termed Febre Negra de Lábrea (Labrea Black Fever) [7,8,9]. It manifested in localized microepidemics, predominantly affecting children and young adults, and was associated with distinct histopathological lesions [7,9]. Clinical presentation included hemorrhagic digestive symptoms, neurological manifestations, and rapid progression to death. Cases were primarily identified in the Purus and Juruá river regions [10].

The hyperendemicity of HBV in this area was already evident in early studies; Bensabath et al. found a 17.9% positivity rate for the Au antigen (HBsAg) among residents with a history of hepatitis along the Purus River in 1969–1970 [11]. This scenario was later confirmed by a study in the municipality of Boca do Acre (1979–1984), which found that 60% of the local population had been infected with HBV and 6.9% were chronic carriers [12]. Another study, conducted in 1985 in other parts of the Amazonas state, revealed a similar high exposure, finding 16.7% HBsAg positivity (96/574), and 33 of these 96 carriers (34.4%) were also co-infected with the hepatitis delta virus [13].

Given the severity of the disease and the high endemicity of these viruses, the Brazilian government implemented a hepatitis B vaccination program in 1989, initially targeting cities with high prevalence in the state of Amazonas [14,15]. Among these, the city of Boca do Acre conducted a study to evaluate the immunogenicity and efficacy of the hepatitis B vaccine, immunizing all children under the age of ten. Vaccination efforts were subsequently expanded, and by 1998, the hepatitis B vaccine had been incorporated into the national immunization program [16,17]. Today, the vaccine is universally available for both children and adults throughout Brazil [17].

The introduction of hepatitis B vaccination has contributed to a global decline in HBV and HDV prevalence. In Brazil, the Brazilian National Hepatitis A, B, and C Survey (2007–2008) reported a reduction in HBV prevalence across all regions, with HBsAg positivity rates below 1% [18]. However, since this survey was limited to urban capitals, it may not accurately reflect the epidemiological scenario in hyperendemic rural areas, where HBV historically exhibited higher transmission rates. Conducting seroepidemiological surveys in rural and riverside areas is challenging due to logistical and accessibility issues. Thus, this study aimed to assess the current prevalence of hepatitis B and delta viruses across both urban and rural/riverside communities within a historically high-prevalence area and to evaluate the impact of hepatitis B vaccination in the region.

## 2. Materials and Methods

### 2.1. Study Design

A population-based, cross-sectional, observational study was conducted from 10 April to 9 May 2023, in the municipality of Boca do Acre, Amazonas, Brazil. Following the initial screening phase, a second field visit was undertaken in August 2024. The objectives of this follow-up were to deliver individual results to participants by a physician, to collect additional biological samples from all serologically confirmed positive cases to confirm the initial serological findings, and to collect samples from all household contacts of the positive index cases.

According to the latest census (2020), Boca do Acre had an estimated population of 34,635 inhabitants, with a territorial area of 21,938.583 km^2^ and a population density of 1.62 inhabitants/km^2^ [19]. The city lies at the confluence of the Purus and Acre rivers, giving it its name, “Boca do Acre” (Mouth of the Acre), and has both an urban population and rural/riverside communities. The rural/riverside population is distributed across small villages along the rivers. Many of these communities are remnants of former rubber extraction sites from the Amazon’s rubber boom era. Few communities are accessible by road; most can only be reached by boat, making river travel essential for trade, travel, and supplies. The region’s economy relies on subsistence farming, fishing, small-scale livestock farming, and limited extractive activities, such as Brazil nut harvesting. Livestock production has also increased in the region, driven by land-use changes and growing demand.

A representative sample size was calculated using a stratified approach to ensure coverage of both urban and rural/riverside areas, based on demographic data from the Brazilian Institute of Geography and Statistics (IBGE) and the Health Department of the Boca do Acre Municipal Government.

In the urban area, the sample size for each neighborhood was evenly distributed among the Community Health Agents to ensure random and representative sampling. To avoid overrepresentation, no more than two individuals per household were included. Sampling was conducted across all five Basic Health Units in the municipality, covering the following neighborhoods: Platô do Piquiá, Centro, Macaxeiral, Praia do Gado, and São Paulo. Additionally, house visits were conducted in all neighborhoods with the assistance of the community’s agents, including individuals with limited mobility, thereby ensuring broader population coverage.

**Figure 1:**
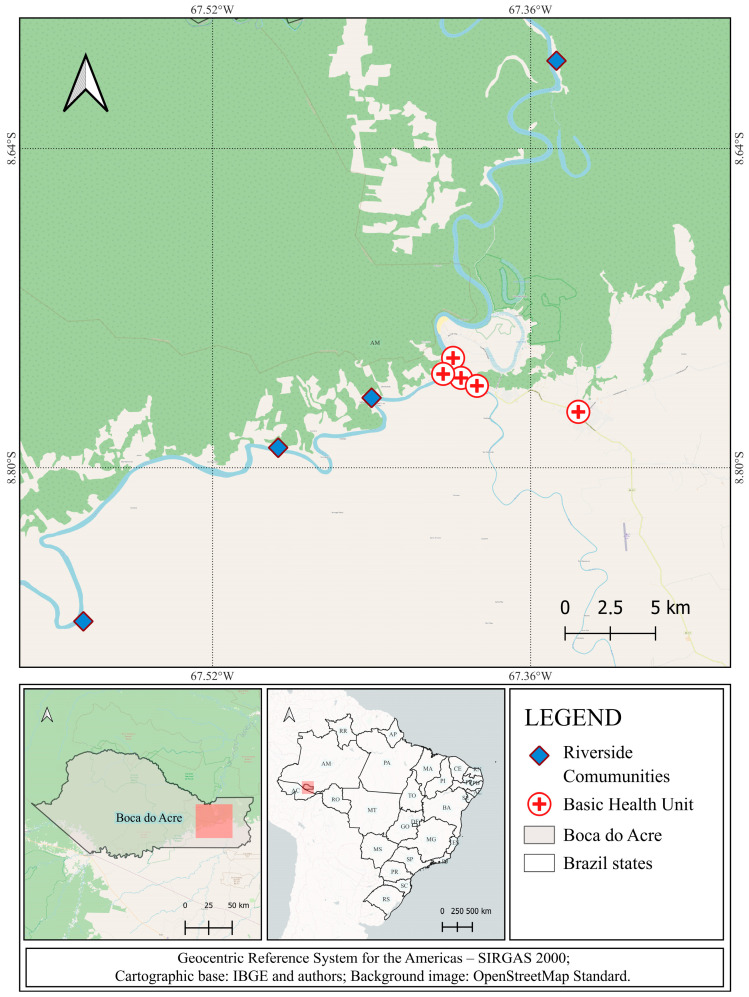
Location of the study area and distribution of sampling sites in Boca do Acre, Brazil.

Four riverside communities (Independência, Cajueiro, Nova Vida, and Bem Posta) were accessed by boat. Additional riverside communities were reached during an annual campaign conducted by the municipality’s fluvial Basic Health Unit. This unit provides medical consultations, dental care, social assistance, and vaccinations to riverside communities along the Purus and Acre Rivers during the flood season. The research team accompanied the fluvial Basic Health Units to collect samples in these communities. The geographic distribution of the sampled urban neighborhoods and rural communities is illustrated in Figure 1.

Each participant answered a questionnaire regarding socioenvironmental and epidemiological characteristics to evaluate possible risk factors, such as previous surgery, history of hepatitis and jaundice, tattoos, blood transfusion, injection drug use, and vaccination history. Whole blood samples were collected after obtaining signed informed consent. The serum was separated by centrifugation, and aliquots were prepared for both serological and molecular testing. The samples were stored at −20 °C and transported on dry ice to the laboratory for analysis.

### 2.2. Serology

All samples were tested for the following HBV serological markers:

- HBsAg (Biolisa HBsAg, BIOLISA, Guayaquil, Ecuador; HBsAg ELISA, Wiener Lab, Rosario, Argentina);
- Total anti-HBc (Monolisa Anti-HBc PLUS, BIO-RAD, California, CA, USA; AFG Bioscience, Illinois, IL, USA; anti-HBc ELISA, Wiener Lab, Rosario, Argentina);
- Quantitative anti-HBs (Biolisa anti-HBs, Bioclin, Minas Gerais, Brazil).

Samples with evidence of current or past HBV infection were further tested for total antibodies to hepatitis delta antigen (HDV Ab; Dia.Pro, Milan, Italy; Elabscience, Wuhan, China). Additionally, HBeAg and anti-HBe (HBe Ag&Ab ELISA; Dia. Pro, Milan, Italy) were assessed in HBsAg-positive samples and anti-HBc-positive alone samples (anti-HBs < 10 mIU/mL and HBsAg-negative). Inconclusive results were retested using alternative assays. All serological markers were analyzed by enzyme immunoassay following the manufacturer’s protocols. Individuals testing positive for hepatitis B or D were referred to tertiary care for further evaluation and management.

### 2.3. Molecular Biology

HBV-DNA quantification was performed for HBsAg-positive samples and anti-HBc-positive-only samples using the Cobas HBV assay on the Cobas 4800 system (Roche Diagnostics, Mannheim, Germany). This automated system performs both extraction and qPCR, with a linear range of 10–1.0 × 10^9^ IU/mL. For HDV-RNA detection, an in-house test was performed using the SuperScript™ III One-Step RT-PCR System (Invitrogen, Carlsbad, CA, USA) as described [20].

### 2.4. Statistics

Differences in prevalence across sex, age, and area were analyzed using Fisher’s Exact Test, with statistical significance set at 0.05. All statistical computations were conducted using software Jamovi version 2.6 (The JamoviProject, 2025 [21]).

### 2.5. Ethical Considerations

This study was approved by the Research Ethics Committee of the Evandro Chagas Institute (IEC) (CAAE no. 61170222.3.0000.0019). Before enrollment, all participants or their legal guardians (for minors under 18 years of age) received a detailed explanation of the study’s objectives, procedures, and implications. Written informed consent was obtained from each participant and their guardian, if the participant was under 18 years of age. The inclusion criteria consisted of individuals aged 6 months or older who were residents of the municipality. Individuals who did not reside in the area or refused to sign the consent form were excluded from the study.

## 3. Results

A total of 1102 serum samples were initially collected. After excluding two samples due to repeatedly inconclusive results, 1100 samples were analyzed. Among the participants, 662 (60.2%) were female, with a mean age of 35.1 years (SD = 21). Sociodemographic and sample characteristics are summarized in Table 1.

HBsAg was detected in 16 individuals (1.5%). A total of 340 participants (30.9%) exhibited serological markers indicative of previous hepatitis B infection (anti-HBs and anti-HBc positive). Additionally, 113 (10%) had isolated anti-HBc positivity (HBsAg and anti-HBs negative). The anti-HD marker was positive in 12 individuals, four of whom were also HBsAg-positive (25%). No HBsAg-positive cases were found in individuals aged 0–19 years and 90–99 years. Importantly, no statistically significant differences in HBV marker prevalence were observed according to sex or area of residence, indicating that the observed epidemiological patterns were consistent across these demographic groups. The distribution of HBsAg, total anti-HBc, and anti-HBs seroprevalence, stratified by sex, age group, and district of residence, is summarized in Table 2.

The analysis of risk factors among the 16 HBsAg-positive individuals revealed that a history of surgery was common, reported in 50% (6/12) of the cases. Other significant factors included inconsistent condom use, a personal history of hepatitis (73.3%, 11/15), contact with infected family members, especially mother, siblings, and cousins (38.5%, 5/13), and having tattoos or a history of injectable medication use (3 cases each). Notably, no cases of blood transfusion, hemodialysis, or injection drug use were reported.

Analysis by year of birth, reflecting the timing of the introduction of the hepatitis B vaccine, revealed a distinct pattern in the distribution of markers. As detailed in Table 3, the prevalence of current infection (HBsAg) was lowest among those eligible for universal infant vaccination (<25 years, 0.2%). In contrast, exposure to HBV (anti-HBc positive) was markedly high in the older, unvaccinated participants (>45 years, 82.5%) compared to the youngest group (4.1%).

Among the 16 HBsAg-positive participants, all were HBeAg-negative, and 13 were anti-HBe-positive. HBV-DNA testing was performed on 15 samples, and the virus was detected in 11 (73.33%) carriers, with viral loads ranging from <10 IU/mL to 3.7 × 10^6^ IU/mL. Four individuals had undetectable viral loads.

Anti-HD prevalence among HBsAg-positive patients was 25% (4/16); in two of them, HDV RNA was detected, indicating active hepatitis delta infection. Among anti-HBc-positive patients, the prevalence of anti-HD was 1.7%. In all of them, the HDV RNA was undetectable.

Of the anti-HDV-positive patients, nine had sufficient sample volume for HBV viral load testing. Of these, only three tested positive, exhibiting very low HBV DNA levels (range: 23.2–156 IU/mL). HDV-RNA was detected in 2 out of 4 anti-HDV-positive and HBsAg-positive individuals.

Among 113 samples with isolated anti-HBc positivity (HBsAg-negative and anti-HBs < 10 mIU/mL), viral load testing was performed in 108 samples. HBV DNA was detectable in two cases: one at a level below the lower limit of quantification (<10 IU/mL) and the other at 16.2 IU/mL.

From the initial group of 16 HBsAg-reactive individuals, follow-up samples were obtained from 13 subjects; two individuals could not be located, and one was deceased. Of the 13 subjects followed, two exhibited seroconversion, characterized by the loss of HBsAg and the development of anti-HBs seropositivity. The remaining individuals remained HBsAg-positive. Follow-up samples were also obtained from the two individuals with isolated anti-HBc (HBsAg-negative, anti-HBc-positive, and anti-HBs-negative) and a detectable viral load. They exhibited the same serological profile, and the viral load was undetectable in the subsequent sample. Samples were collected from 26 household contacts of the 18 positive individuals (16 HBsAg-reactive and 2 with isolated anti-HBc). This group of contacts comprised eight sexual partners, 15 offspring, one sibling, one mother, and one granddaughter.

The serological assessment of household contacts revealed the following distribution: among sexual partners (*n* = 8), one possessed protective anti-HBs titers (>10 mIU/mL), five had markers of a past resolved infection, and two were susceptible; among offspring (*n* = 15), five possessed protective anti-HBs titers (>10 mIU/mL), two had markers of a past resolved infection, and eight were susceptible; the one sibling presented with a current HBV and a past HDV infection (HBsAg positive, anti-HDV positive, HBV-DNA detected, HDV-RNA not detected); the one mother had markers of a past HBV resolved infection; and the one granddaughter was susceptible.

## 4. Discussion

The data presented in this study demonstrate a significant reduction in the prevalence of hepatitis B surface antigen (HBsAg) in the population of Boca do Acre, reflecting the positive impact of the hepatitis B vaccination program. A comparison between seroepidemiological data from the pre-vaccination era (1979–1984) [12] and the present study shows a marked decrease in HBsAg prevalence rates, from 6.9% to 1.5%, while the overall marker of exposure (anti-HBc total) has decreased from approximately 60% to 30.9%, indicating a substantial shift from high endemicity to low endemicity [12].

Hepatitis B vaccination is the most critical tool to control HBV. Several countries have successfully implemented HBV vaccination programs, with marked reduction in carrier rates and the associated complications [22]. One of the most successful cases is Taiwan. This country implemented the HBV vaccine as early as 1984 and now achieves an impressive 97.7% HBV vaccine coverage, reducing HBsAg prevalence from 9.8% in the pre-vaccination period to 0.5% 30 years after the immunization program’s implementation [23,24].

The impact of the HBV vaccine has been demonstrated over recent years in several countries. In the USA, an extended study follow-up has shown substantial effectiveness against HBV infection, with protection maintained for 20 years [25]. In China, where nearly 50% of the Chinese population has a history of HBV infection [26], the implementation of the vaccine in 1992 resulted in a significant reduction in HBsAg carriers from 10% to <1% in children after 2 decades [27].

In our study, one particularly relevant finding was the absence of chronic hepatitis B (HBsAg-positive) cases among children and adolescents under 20 years of age. This aligns with the World Health Organization’s (WHO) global hepatitis B elimination goal, which aims for HBsAg prevalence to be below 0.1% among children under five [28]. Similar results were reported in Peru [29], while Colombia still reported a 0.5% HBsAg prevalence in this age group [30]. Although the number of pediatric participants in our study was limited, likely due to parental hesitancy regarding blood draws from asymptomatic children, the findings suggest a successful interruption of vertical and early childhood transmission. This is a significant achievement, given the higher likelihood of chronic infection in children under five years of age.

This success is closely tied to the prevention of Mother-to-Child Transmission (MTCT), a cornerstone of the WHO’s elimination strategy [31]. The strategy includes hepatitis B birth dose and infant vaccination, testing of pregnant women, and antiviral treatment for those who are eligible [31]. Supporting this approach, a study from Taiwan provided real-world evidence on the impact of maternal antiviral prophylaxis in reducing MTCT [32].

Our findings in Brazil illustrate the profound effect of hepatitis B vaccination on the infection’s natural history. Anti-HBc seroprevalence, a marker of cumulative exposure, showed a striking inverse relationship with the introduction of universal immunization, increasing from 4.1% among individuals aged ≤25 years (those born after the implementation of HBV vaccine in Brazil) to 82.5% in those ≥45 years. This pattern reflects a clear epidemiological transition from a pre-vaccination era characterized by high endemicity to a contemporary era marked by significant reductions in viral transmission. A summary of the history of hepatitis B vaccination in Brazil is presented in Table 4.

Vaccination status, as indicated by the presence of isolated anti-HBs, a serological marker of vaccine-induced immunity, further supports this interpretation. A significant age gradient was observed (*p* < 0.001), with vaccination coverage highest in the youngest group (31.2%) and declining progressively to 10.5% in the oldest group. This gradient aligns closely with the declining burden of HBV exposure across generations.

Notably, the distribution of HBsAg, indicating active chronic infection, reveals a more nuanced pattern. Contrary to expectations, the highest prevalence was found among adults aged 26–44 years (2.4%), rather than in the oldest group (2.0%), which had the highest historical exposure. This discrepancy may be attributed to mortality related to chronic HBV infection among older individuals, who face an elevated lifetime risk of fatal complications such as decompensated cirrhosis and hepatocellular carcinoma.

Thus, the 26–44-year age group emerges as a high-risk “transition generation”, old enough to have experienced substantial HBV exposure before widespread vaccine availability (as reflected in their low vaccination rate of 28.6%), yet young enough to still carry a high prevalence of chronic infection without having yet experienced whole disease progression. These results highlight the urgent need for targeted screening, linkage to care, and treatment initiatives in this age group to reduce future complications. Meanwhile, the low rates of both exposure and active infection among the younger group, coupled with higher vaccine coverage, provide compelling evidence of the effectiveness of universal childhood vaccination against HBV in Brazil.

Beyond the reduction in prevalence, the character of persistent infection itself appears to have evolved. This is evidenced by a stark difference in the detection of HBeAg-positive cases, which accounted for nearly 50% in 1979 [14], whereas none were detected in the current sampling. Additionally, the viral loads observed were extremely low. A clinical evaluation of chronic carriers would be interesting to assess whether these findings correlate with disease severity. Furthermore, it is essential to address the occurrence of mutations in the preCore/Core region, which have been associated with HBeAg negativity, immune escape, and persistent hepatitis [33].

The occurrence of isolated anti-HBc positivity adds complexity to the current epidemiological understanding of hepatitis B. The current study demonstrated that 113 out of 1100 subjects (10.3%) had isolated anti-HBc positivity. Several factors may account for this finding, including resolved infection (with or without waning anti-HBs), false seroreactivity, or clinically relevant occult hepatitis B infection (OBI). Among these cases, HBV DNA was detectable in two individuals (1.7% of anti-HBc-only samples), with low-level viremia (<20 IU/mL) as reported in the literature, confirming OBI in a subset of patients [34]. These findings highlight the heterogeneity of isolated anti-HBc positivity. In addition, our data demonstrated that the proportion of isolated anti-HBc positivity increased with advancing age, which can be explained by the progressive decline of anti-HBs over time. Although viral loads were low, the presence of detectable HBV DNA underscores the potential risk of reactivation, particularly in immunocompromised hosts. Our data support the current recommendation of HBV DNA screening in high-risk anti-HBc-positive patients, even in the absence of overt liver disease.

To further explore transmission dynamics in this new low-endemicity context, we analyzed serological analysis of household contacts, stratified by relationship to the index case, which revealed distinct patterns of hepatitis B exposure and susceptibility. Among conjugal partners, the majority exhibited evidence of past resolved infection (5/8; 62.5%), one was immune, likely from vaccination, and two (25%) remained susceptible; no active infections were detected. In contrast, the profile for offspring was marked by a high rate of susceptibility, with five immune individuals and two having evidence of past infection. Active infection was identified in one sibling, and a past infection was found in one mother. The presence of a susceptible granddaughter underscores the potential for intergenerational transmission within the household. These findings highlight the household as a critical environment for HBV transmission dynamics and clearly demonstrate that risk, whether of prior exposure, current infection, or susceptibility, is strongly influenced by the specific familial relationship to the index case. This evidence strongly advocates for the comprehensive screening and subsequent vaccination of all susceptible household contacts. Also, the analysis of the risk factors of the 16 HBsAg individuals revealed that surgery, intrafamilial contact, and unprotected sex were the primary risk factors for HBV transmission. However, these findings must be interpreted with caution, as the data are based on participant self-reporting.

The vaccination program’s impact also extends to hepatitis D virus (HDV) coinfection. In our study, 12 out of 1100 samples tested positive for anti-HDV, including four that were also HBsAg-positive, confirming HBV/HDV coinfection. Notably, HDV RNA was detected in two of these coinfected cases, demonstrating active HDV infection. The remaining eight anti-HDV-positive cases occurred in HBsAg-negative individuals, suggesting that these individuals had resolved HBV infections with past HDV exposure.

These findings highlight that HDV infection remains a persistent concern, particularly among adults over 30 years of age. The presence of anti-HDV in HBsAg-negative individuals highlights the importance of testing for HDV antibodies, regardless of HBsAg status, as this can aid in identifying past infections and better define the actual epidemiological burden of HDV.

Given HDV’s dependence on HBV, its declining prevalence aligns with global trends in HBV control. However, the detection of both active (RNA-positive) and resolved (anti-HDV+/HBsAg−) infections emphasizes that HDV still warrants clinical attention. Patients with confirmed HDV replication require close monitoring due to the risk of accelerated liver disease. At the same time, those with isolated anti-HDV may benefit from further assessment to rule out occult infection or advanced fibrosis from prior exposure.

Although current guidelines recommend systematic HDV screening, including routine anti-HDV testing for all HBsAg-positive individuals and selective testing for high-risk groups, these measures are rarely implemented. This gap in practice highlights the crucial importance of population-based serosurveys in accurately determining the true prevalence of HDV. Our work demonstrates the profound impact of universal hepatitis B vaccination, which has drastically reduced HDV circulation and created a generational break in transmission, evidenced by the complete absence of infections in individuals above 30 years of age. This markedly contrasts with pre-vaccination era studies, which reported a widespread prevalence across all age groups.

However, full implementation of existing screening recommendations is now crucial. It would significantly improve detection rates by capturing both active (HDV RNA-positive) and resolved infections, highlighting the persistent clinical relevance of HDV. Our findings, which identified active HDV replication and a substantial susceptible population (33.6%), emphasize that this gap represents a missed opportunity for early diagnosis. It also underscores the continued vulnerability to future coinfection and the enduring importance of HBV vaccination programs. Together, these results suggest that while HDV prevalence is declining in parallel with HBV control, comprehensive testing remains essential to identify at-risk individuals, guide clinical management, and monitor epidemiological trends in the post-vaccination era. In summary, while HDV prevalence has declined in tandem with successful HBV control, it remains a non-negligible infection that requires targeted screening efforts.

A notable limitation of our study was that, unfortunately, few individuals brought their vaccination records, which hindered the ability to accurately correlate their immunization history with serological findings regarding hepatitis B vaccine doses. This limitation was particularly relevant for HBV marker-negative cases, where individuals presumed to be protected often could not recall completing the whole vaccine series. The low rate of individuals with protective levels of anti-HBs (≥10 mIU/mL) observed in our study (23.7%) further complicates this assessment. A survey of scholars from Cameron observed that more than 76% of vaccinated individuals displayed negative anti-HBs [35]. However, it is essential to note that while antibody titers may wane over time, anamnestic immune memory likely maintains protection [36]. Beyond waning immunity, current HBV vaccines face broader challenges, including multi-dose regimens, reduced efficacy in adults, and limited access in low-income countries and rural localities [22]. To overcome these challenges, researchers are investigating next-generation solutions, including vaccines with more immunogenic adjuvants, novel delivery devices like Uniject, a prefilled, single-use syringe, and alternative administration routes (intradermal, oral, nasal) [37]. However, further studies are needed to thoroughly evaluate the safety and performance profile of these promising new alternatives before they can be widely implemented.

## 5. Conclusions

Brazil’s HBV vaccination program exemplifies a public health achievement, which, despite suboptimal coverage, has been effective in eliminating vertical transmission and reducing the number of chronic carriers. This survey confirms that following its introduction in Boca do Acre, the prevalence of HBV and HDV has shifted from very high to low endemicity. However, persistent challenges such as occult hepatitis B, susceptible household clusters, and the legacy of HDV coinfection underscore that elimination remains an ongoing mission. Persistent efforts to optimize coverage, integrate enhanced vaccination record systems, adhere to HDV screening guidelines, and target public health interventions for household contacts will ensure continued reductions in HBV and HDV burden, safeguarding future generations.

## Figures and Tables

**Table 1:** Demographic characteristics of the study participants.

	N	%
Area		
Urban	849	77.2
Rural/Riverside	251	22.8
Age group		
0–4	42	3.8
5–9	80	7.3
10–14	100	9.1
15–19	95	8.6
20–29	162	14.7
30–39	174	15.8
40–49	165	15.0
50–59	129	11.7
60–69	81	7.4
70–79	43	3.9
>80	29	2.6
Sex		
Female	662	60.2
Male	438	39.8
Total	1100	100

**Table 2:** Seroprevalence of hepatitis B virus markers by area, age group, and sex.

	N	HBsAg +	Total a-HBc + and a-HBs > 10 mUI/mL	Total a-HBc Positive Alone (HBsAg and a-HBs Negative)	a-HBs > 10 mUI/mL (with HBsAg and a-HBc Negative)	All Negative	*p* Value
		N (%)	N (%)	N (%)	N (%)	N (%)	
Area							
Urban	849	12 (1.4)	247 (29.1)	86 (7.8)	208 (24.5)	296 (34.9)	0.150
Rural/Riverside	251	4 (1.6)	93 (37.1)	27 (2.5)	53 (21.1)	74 (29.5)	
Age group							
0–4	42	0	0	0	29 (69.0)	13 (31.0)	<0.001
5–9	80	0	1 (1.3)	0	30 (37.5)	49 (61.3)	
10–14	100	0	1 (1.0)	0	25 (25.0)	74 (74)	
15–19	95	0	0	0	21 (22.1)	74 (77.9)	
20–29	162	1 (0.6)	18 (11.1)	11 (6.8)	39 (24.1)	93 (57.4)	
30–39	174	5 (2.9)	63 (36.2)	15 (8.6)	60 (34.5)	31 (17.8)	
40–49	165	5 (3.0)	94 (57.0)	23 (13.9)	28 (17.0)	15 (9.1)	
50–59	129	2 (1.6)	79 (61.2)	23 (17.8)	13 (10.1)	12 (9.3)	
60–69	81	1 (1.2)	48 (59.3)	21 (25.9)	8 (9.9)	3 (3.7)	
70–79	43	1 (2.3)	23 (53.5)	11 (25.6)	4 (9.3)	4 (9.3)	
80–89	21	1 (4.8)	12 (57.1)	6 (28.6)	4 (4.8)	1 (4.8)	
90–99	8	0	1 (12.5)	3 (37.5)	3 (37.5)	1 (12.5)	
Sex							
Female	662	10 (1.5)	201 (30.4)	61 (9.2)	160 (24.2)	230 (34.7)	0.600
Male	438	6 (1.4)	139 (31.7)	52 (11.9)	101 (23.1)	140 (32.0)	
Total	1100	16 (1.5)	340 (30.9)	113 (10.3)	261 (23.7)	370 (33.6)	

**Table 3:** Seroprevalence of hepatitis B markers by age group and vaccination eligibility groups.

Age Group	N	HBsAg + (%)	*p*	a-HBc + (%)	*p*	a-HBs + Isolate (%)	*p*
<25 years ^†^	417	1 (0.2)	0.015	17 (4.1)	<0.001	130 (31.2)	<0.001
26–44 years ^‡^	329	8 (2.4)		159 (48.3)		94 (28.6)	
>45 years ^§^	354	7 (2.0)		292 (82.5)		37 (10.5)	
Total	1100	16		468		261	

^†^ Group primarily eligible for universal infant vaccination since 1998. ^‡^ Group eligible for infant vaccination (under 10 years) since 1989. ^§^ Group unexposed primarily to the hepatitis B vaccine during childhood. Note: Participants are stratified by year of birth based on age at serum collection (2023) and the introduction of the hepatitis B vaccine in Brazil (nationwide in 1998; in specific Amazonas municipalities in 1989).

**Table 4:** Summarized history of national hepatitis B vaccination schedules in Brazil. 1989–2015.

Year	Localities	Age	Doses
1989	For high HBV prevalence cities in Amazonas state	Under 10 years old	BD + 2 HB or 3 HB
1991	For all cities in Amazonas state	Under 1 year old	BD + 2 HB or 3 HB
1992	Vaccination expanded to the entire Legal Amazon, Paraná, Espírito Santo, Santa Catarina, and the Federal District.	Under 5 years old	BD + 2 HB or 3 HB
1998	For all states of Brazil	Under 1 year old	BD + 2 HB or 3 HB
	Legal Amazon, Paraná, Espírito Santo, Santa Catarina, and the Federal District	Under 15 years old	BD + 2 HB or 3 HB
2012	For all states of Brazil	Under 7 years old	BD + 3 pentavalent or 3 HB
		Above 7 years to 29 years	3 HB
2015-until now	For all states of Brazil	All age groups	BD + 3 doses pentavalente or 3 HB

Notes: BD + 2 HB (Birth dose and two doses of hepatitis B vaccine at 0, 1, and 6 months). 3 HB: 3 doses of hepatitis B vaccine at 0, 1, and 6 months). BD + three pentavalent (Birth dose and three doses of pentavalent vaccine at 0 (BD), 2, 4, and 6 months).

## Data Availability

The original contributions presented in this study are included in the article. Further inquiries can be directed to the corresponding author.

## Abbreviations

The following abbreviations are used in this manuscript:

HBV	hepatitis B virus
HDV	hepatitis D virus
HBsAg	hepatitis B surface antigen
anti-HBc	antibody to hepatitis B core antigen
anti-HBs	antibody to hepatitis B surface antigen
anti-HDV	antibody to hepatitis D virus
HBV-DNA	Hepatitis B Virus DNA
HDV-RNA	Hepatitis D Virus RNA
IEC	Evandro Chagas Institute
CAAE	Certificate of Ethical Appreciation Presentation
IBGE	Brazilian Institute of Geography and Statistics
HBeAg	hepatitis B *e* antigen
anti-HBe	antibody to hepatitis B *e* antigen
OBI	occult hepatitis B infection
COVID-19	CoronaVirus Disease 2019
WHO	World Health Organization
MTCT	Mother-to-Child Transmission
